# Investigating Hybrid PLGA-Lipid Nanoparticles as an Innovative Delivery Tool for Palmitoylethanolamide to Muscle Cells

**DOI:** 10.3390/pharmaceutics17111412

**Published:** 2025-10-30

**Authors:** Eleonora Maretti, Susanna Molinari, Sonia Partel, Beatrice Recchia, Cecilia Rustichelli, Eliana Leo

**Affiliations:** 1 Department of Life Sciences, University of Modena and Reggio Emilia, Via Campi 103, 41125 Modena, Italy; eleonora.maretti@unimore.it (E.M.); partelsonia@gmail.com (S.P.); cecilia.rustichelli@unimore.it (C.R.); 2 Department of Life Sciences, University of Modena and Reggio Emilia, Via Campi 287, 41125 Modena, Italy; susanna.molinari@unimore.it (S.M.); beatricerecchia9@gmail.com (B.R.)

**Keywords:** nanocarriers, muscle cells, stearic acid, gelucire, PEA, myoblast, sarcopenia

## Abstract

**Background/Objectives:** Palmitoylethanolamide (PEA) is an endogenous lipid mediator with endocannabinoid-like activity. Despite its therapeutic potential in muscle-related inflammatory disorders, including sarcopenia, its clinical use is limited by poor solubility and bioavailability. To overcome these issues, we developed hybrid nanoparticles combining poly(lactic-co-glycolic acid) (PLGA) and lipids to enhance PEA encapsulation and ok delivery. **Methods**: PEA-loaded hybrid nanoparticles (PEA-Hyb-np) were produced via a modified single-emulsion solvent evaporation method using stearic acid and Gelucire^®^ 50/13 as lipid components. Characterization included particle size, morphology, PDI, and zeta potential, as well as DSC, FT-IR, and XRD analyses. For the biological evaluation in a C2C12 myoblasts cell culture, coumarin-6-labeled nanoparticles were employed. **Results**: PEA-Hyb-np showed mean particle sizes of ~150 nm, with internal lipid–polymer phase separation. This structure enabled high encapsulation efficiency (79%) and drug loading (44.2 mg/g). Drug release in physiological and non-physiological media was enhanced due to drug amorphization, confirmed by DSC, FT-IR, and XRD analyses. Cytocompatibility studies showed no toxicity and improved cell viability compared to unloaded nanoparticles. Cellular uptake studies by confocal microscopy and flow cytometry demonstrated efficient and time-dependent internalization. **Conclusions**: PEA-Hyb-np represent a promising delivery platform to improve the solubility, bioavailability, and therapeutic efficacy of PEA for muscle-targeted applications.

## 1. Introduction

Palmitoylethanolamide (PEA) is a naturally occurring lipid amide that belongs to the N-acylethanolamine family. It has attracted increasing scientific interest due to its potent anti-inflammatory, analgesic, and neuroprotective properties, which are mediated through multiple molecular pathways, including modulation of mast cell activation, interaction with peroxisome proliferator-activated receptor alpha (PPAR-α), and indirect regulation of the endocannabinoid system [1,2]. This compound has been extensively investigated in preclinical and clinical settings for a variety of pathological conditions, including chronic pain syndromes, neurodegenerative diseases, and inflammatory disorders [3,4]. More recently, PEA has been proposed as a promising therapeutic candidate for muscle-related inflammatory conditions such as sarcopenia, an age-related progressive and debilitating disorder characterized by the loss of skeletal muscle mass and strength [5,6,7].

Despite its well-documented pharmacological potential, the clinical translation of PEA as drug has been hindered by significant biopharmaceutical limitations, most notably its poor aqueous solubility and limited oral bioavailability. These drawbacks are attributed to its highly lipophilic nature and rapid metabolic degradation, which collectively reduce systemic absorption and therapeutic efficacy [8,9]. Consequently, innovative drug delivery strategies are critically needed to overcome these pharmacokinetic limitations and enable more effective tissue targeting and sustained release profiles.

Nanotechnology-based delivery systems have emerged as powerful tools to improve the solubility, stability, and bioavailability of hydrophobic drugs like PEA. Among these, poly (lactic-co-glycolic acid) (PLGA)-based nanoparticles represent a well-established platform due to their biodegradability, biocompatibility, and approval by regulatory agencies such as the Food and Drug Administration (FDA) and the European Medicines Agency (EMA) [10,11]. PLGA nanoparticles offer the potential to encapsulate a wide range of bioactive molecules and modulate their release kinetics in a controlled manner. However, their application to highly lipophilic compounds such as PEA remains challenging. Specifically, the encapsulation of lipid-like molecules in PLGA matrices is often inefficient, primarily due to poor drug-polymer compatibility and drug expulsion during nanoparticle formation [12,13].

To address this issue, hybrid nanoparticle systems have gained attraction as an alternative approach. These systems combine the structural benefits of polymeric nanoparticles with the solubilizing and stabilizing properties of lipid components, enabling improved encapsulation of hydrophobic drugs and better interaction with biological membranes [14,15]. Several recent studies have demonstrated the enhanced performance of lipid-polymer hybrid nanoparticles in terms of drug loading, release kinetics, and cellular uptake, especially in the context of oral, pulmonary, and transdermal delivery [16,17]. However, their application to skeletal muscle-targeted therapies remains underexplored.

In this study, a novel hybrid delivery platform was developed by integrating lipid excipients, namely stearic acid and Gelucire^®^ 50/13, into PLGA-based nanoparticles to effectively encapsulate PEA. Stearic acid was selected due to its biocompatibility and known ability to form stable hydrophobic interactions with PEA, thereby enhancing its affinity for the polymer matrix [18]. Gelucire^®^ 50/13, a lipid excipient modified with polyethylene glycol (PEG) chains, was incorporated to provide surfactant properties and further stabilize the hybrid structure. The resulting formulation was designed to overcome the encapsulation inefficiencies observed in conventional PLGA systems while preserving the favorable characteristics of polymeric nanoparticles.

The objectives of this work were threefold: (i) to optimize the formulation parameters to achieve high PEA encapsulation efficiency and stable colloidal properties; (ii) to characterize the physicochemical properties of the nanoparticles, including size distribution, zeta potential, morphology, drug loading, and release kinetics; and (iii) to perform preliminary biological evaluation using murine C2C12 myoblasts to assess cytotoxicity and cellular uptake. This proof-of-concept study lays the foundation for further development of PEA-loaded hybrid nanoparticles as a potential delivery strategy for the treatment of muscle-associated inflammatory conditions, offering improved bioavailability and targeted intracellular delivery.

## 2. Materials and Methods

### 2.1. Materials

Palmitoylethanolamide (PEA) was kindly provided by Innexus Nutraceuticals (Nijmegen, The Netherlands). Stearic acid, Pluronic^®^ F68, and Span^®^ 85 were purchased from Sigma-Aldrich (St. Louis, MO, USA). Poly(lactic-co-glycolic acid) (PLGA; Resomer^®^ RG 504) was obtained from Evonik Industries (Essen, Germany). Gelucire^®^ 50/13 was a generous gift from Gattefossé (Saint-Priest, Cedex, France). Sodium taurocholate (from bovine bile) was obtained from Tokyo Chemical Industry (Tokyo, Japan) and Coumarin-6 from ThermoFisher Scientific (Monza, Italy).

For cell culture studies, the C2C12 mouse myoblast cell line was provided by Dr. M. Buckingham (Institut Pasteur, Paris, France). Dulbecco’s Modified Eagle Medium (DMEM) was obtained from EuroClone (Milan, Italy). L-glutamine, paraformaldehyde, and phosphate-buffered saline (PBS) were purchased from Sigma-Aldrich. Trypsin-EDTA and penicillin–streptomycin (P/S) were obtained from Lonza (Milan, Italy) and fetal bovine serum (FBS) from Gibco, a brand of Thermo Fisher Scientific (Waltham, MA, USA). Hoechst 33342 was purchased from Thermo Fisher Scientific (Monza, Italy), and Thiazolyl Blue Tetrazolium Bromide (MTT) from PanReac AppliChem ITW Reagents (Milan, Italy). All the other chemicals were of HPLC grade.

### 2.2. Hybrid Lipid-PLGA Nanoparticle Preparation

To explore formulation parameters, a stepwise approach was employed starting from a basic PEA-loaded PLGA nanoparticle system (PEA-PLGA-np), followed by the incorporation of stearic acid (PEA/Ste-PLGA-np) or Gelucire^®^ 50/13 (PEA/Gel-PLGA-np), and culminating in the optimized hybrid formulation containing both stearic acid and Gelucire^®^ 50/13 (PEA-Hyb-np) (Table 1). This optimized PEA-Hyb-np formulation was prepared using a modified solvent evaporation method as illustrated in Figure S1. Briefly, 80 mg of PLGA was dissolved in 3 mL of dichloromethane (DCM). Subsequently, PEA (10 mg), stearic acid (10 mg), Gelucire^®^ 50/13 (80 mg), and Span^®^ 85 (120 mg) were incorporated into the organic phase. This solution was introduced into 10 mL of an aqueous phase containing Pluronic^®^ F68 (0.8%, *w*/*v*) under sonication in an ice bath for 5 min at 70% amplitude (SFX150 Branson, Milan, Italy) and further homogenized using Ultra-Turrax (T-25 basic, Ika Labortechnik, Staufen im Breisgau, Germany) at 24,000 rpm for 15 min.

Organic solvent removal was achieved by magnetic stirring (1000 rpm) for 2 h at room temperature to allow DCM evaporation. The resulting nanoparticle suspension was purified via dialysis using a membrane with 12/14 kDa molecular weight cut-off (MWCO) (Sigma Aldrich, Milan, Italy) against 1 L of Milli-Q water for 30 min. A final volume of 10 mL of purified suspension was collected and stored at 4 °C for further characterization. Unloaded hybrid nanoparticles (U-Hyb-np) were prepared using the same procedure, excluding PEA, while for internalization studies, PEA-Hyb-np was prepared using PEA pre-labeled with Coumarin-6. Labeling was performed by dissolving Naked PEA in ethanol and incorporating Coumarin-6 at 0.1%, *w*/*w*. The ethanol solution was evaporated under reduced pressure to yield a fluorescently labeled PEA powder (Naked PEA_Cum_), which was subsequently used in the nanoparticle preparations to formulate fluorescent PEA-Hyb-np (PEA-Hyb-np_Cum_).

### 2.3. Particle Size, Surface Charge, and Morphology Analysis

Particle size (Z-average), polydispersity index (PDI), and zeta potential were measured by Dynamic Light Scattering (DLS) using a Zetasizer (version 6.12, Malvern Instruments, Worcestershire, UK), equipped with a 4 mW He-Ne laser (633 nm) and DTS software (version 5.0). Measurements were performed on samples diluted 1:100 (*v*/*v*) in deionized water. Reported values represent the mean ± standard deviation from three independent batches.

### 2.4. Transmission Electron Microscopy

The morphology of hybrid nanoparticles was examined using Transmission Electron Microscopy (TEM; Talos F200S G2, Thermo Fisher Scientific, Milan, Italy) operated also in scanning TEM (STEM) mode with High-angle annular dark-field (HAADF) detector. Carbon-coated copper TEM grids were briefly dipped into a 1:10 (*v*/*v*) dilution of PEA-Hyb-np suspension in deionized water. Grids were then air-dried and coated with a thin carbon layer using a Carbon Coater (Balzers CED-010, Oerlikon Balzers, Balzers, Liechtenstein) prior to imaging.

### 2.5. Quantification of PEA by HPLC

The content of PEA in all formulations was quantified using high-performance liquid chromatography (HPLC). The HPLC system consisted of a PU-4180 pump coupled with a UV-4075 UV-Vis detector, and data acquisition was performed using ChromNAV software (Version 2.0, Jasco Corporation, Tokyo, Japan).

Chromatographic separation was carried out on an InertClone OSD reversed-phase column (100 Å, 150 × 4.6 mm, 5 μm particle size), protected by a C18 guard column (4 × 3 mm I.D.) (Phenomenex, Milan, Italy). The mobile phase comprised acetonitrile and water (80:20, *v*/*v*), delivered isocratically at a flow rate of 1.0 mL/min. The column temperature was maintained at 30 °C, with a 10.0 μL injection volume and the detection was performed at 210 nm using UV absorbance.

### 2.6. Determination of Encapsulation Efficiency and Drug Loading

Drug content was determined in terms of encapsulation efficiency (EE, %) and, only for the optimized PEA-Hyb-np, in terms of drug loading (DL, mg/g), For sample preparation, 250 μL of nanoparticle suspension was mixed with 750 μL of acetonitrile to disrupt the particles and extract PEA. The mixture was vortexed for 2 min and subsequently sonicated in an ultrasonic bath for 20 min. The resulting mixture was filtered through a 0.2 μm hydrophobic PTFE membrane filter prior to HPLC analysis.

EE and DL were calculated according to the following equations:

EE = incorporated drug (mg)/initial drug (mg)∙100(1)

DL = incorporated drug (mg)/total mass of particles (g)(2)

### 2.7. Stability Study

An experimental stability study was carried out to assess the colloidal stability of PEA-Hyb-np in aqueous suspension over time. Both PEA-Hyb-np and U-Hyb-np were stored at 4 °C and the size was monitored for 80 days.

In selected days, an aliquot of the suspension was diluted 1:10 (*v*/*v*) in deionized water and analyzed using a Zetasizer to measure particle size and PDI. The experiment was conducted in triplicate using independently prepared batches.

### 2.8. In Vitro PEA Release

The in vitro release of PEA from PEA-Hyb-np was evaluated in two different media: a non-physiological aqueous solution containing 2% *w*/*v* sodium taurocholate, and a physiological phosphate buffer at pH 7.4.

Exactly volume of PEA-Hyb-np suspension were placed into a dialysis membrane with 12/14 kDa MWCO and subsequently immersed in 10 mL of the respective release medium and incubated at 37 ± 1 °C under continuous magnetic stirring. At predetermined time points (1, 2, 4, 6, and 24 h), 500 μL aliquots were withdrawn from the medium. The amount of released PEA was quantified using HPLC, following the chromatographic conditions previously described. After each sampling, an equal volume (500 μL) of fresh pre-warmed release medium was added to maintain a constant volume of 10 mL throughout the experiment. For comparison, Naked PEA was also incubated under the same conditions in both media to assess its solubility and dissolution profile.

### 2.9. Physical State Characterization of PEA

#### 2.9.1. DSC Analysis

Thermal properties of the formulations were evaluated using differential scanning calorimetry (DSC 4000, PerkinElmer, Norwalk, CT, USA). The instrument was calibrated using indium as a standard. Samples were heated from 30 °C to 120 °C at a rate of 10 °C/min under a constant nitrogen flow of 40 mL/min. Analyses were performed on PEA-Hyb-np, U-Hyb-np, individual formulation components (PEA, PLGA, stearic acid, Gelucire^®^ 50/13), and their physical mixtures.

#### 2.9.2. FT-IR Analysis

Fourier transform infrared (FT-IR) spectroscopy was carried out on the same set of samples using a Spectrum Two spectrometer (PerkinElmer, Milan, Italy) equipped with a Universal Attenuated Total Reflectance (ATR) accessory. Samples were directly applied to the ATR crystal surface. Spectra were recorded in the range of 4000–450 cm^−1^ with a spectral resolution of 4 cm^−1^. To optimize the signal-to-noise ratio, 16 scans were averaged per spectrum. Background spectra were collected under identical conditions prior to each sample measurement. All analyses were conducted in triplicate, and representative results are reported.

#### 2.9.3. XRD Scanning Analysis

XRD analysis were recorded by Empyrean III Multicore, using Detector PIXcel3D (Malvern PANalytical, Almelo, The Netherlands) on Naked PEA, PEA-Hyb-np, U-Hyb-np and Physical mixture of all the np components. The analysis was operated in Bragg–Brentano geometry using Cu Kα radiation (λ = 1.540 Å) with a voltage of 40 kV and a current of 40 mA, recollecting the powders on a zero background sample holder. Data acquisition was conducted in the 2theta range 5–60° (minimum step size 2Theta:0.0001) with a time step of 58 s.

### 2.10. In Vitro Cell Analyses: Cell Culture

The C2C12 murine myoblast cell line was originally derived from satellite cells of mouse thigh muscle [19]. C2C12 cells were cultured in high-glucose DMEM supplemented with 2 mM L-glutamine, 1% P/S, and 10% FBS. Cells were maintained at low confluence in a humidified incubator at 37 °C with 5% CO_2_.

### 2.11. Cytotoxicity Test

The cytotoxicity of PEA-Hyb-np was evaluated in comparison with U-Hyb-np and Naked PEA using the MTT assay. C2C12 cells were seeded in 24-well plates at a density of 4000 cells/well in complete growth medium and allowed to adhere for 24 h under standard culture conditions. Following incubation, cells were treated with PEA at final concentrations of 10, 25, or 100 μM, either in the form of PEA-Hyb-np or as Naked PEA. Incubation periods were 2, 14, and 24 h. An equivalent amount of U-Hyb-np (corresponding to the amount used in the PEA-Hyb-np treatments) was administered as a nanoparticle control. All treatments were performed in triplicate.

After the respective incubation periods, MTT solution (5 mg/mL in PBS) was added to each well to reach a final concentration of 0.5 mg/mL. Plates were incubated for 2 h at 37 °C, after which the medium was removed and cells were gently washed with PBS. Dimethyl sulfoxide (DMSO) was then added to each well to solubilize the formazan crystals formed by metabolically active cells. After 15 min of shaking, absorbance was measured at 570 nm using a Multiskan™ FC Microplate Photometer (Thermo Fisher Scientific, Monza, Italy). Cell viability was expressed as a percentage of the absorbance relative to untreated control cells.

### 2.12. Cellular Uptake of Coumarin-6-Labeled Nanoparticles by Flow Cytometric Analysis

The internalization capacity of PEA-Hyb-np_Cum_ was evaluated in C2C12 myoblasts and compared to Naked PEA_Cum_. C2C12 cells were seeded in 6-well plates at a density of 80,000 cells/well and incubated with either PEA-Hyb-np_Cum_ or Naked PEA_Cum_ at a final PEA concentration of 100 μM for 2, 6, and 14 h. The test was performed with six replicates.

Following incubation, cells were washed twice with PBS to remove unbound material, detached by trypsinization using 0.25% Trypsin-EDTA, and centrifuged. Cell pellets were resuspended in PBS and immediately analyzed by flow cytometry using a Coulter Epics XL flow cytometer (Beckman Coulter Inc., Brea, CA, USA) equipped with an argon laser (excitation at 525 nm). For each sample, a minimum of 10,000 events was recorded. Data were expressed as the percentage of fluorescence-positive cells, indicating nanoparticle uptake.

### 2.13. Confocal Microscopy Analysis

For confocal imaging, C2C12 cells were seeded at a density of 1500 cells per well in Nunc™ Lab-Tek™ chamber slides (0.7 cm^2^ surface area; Thermo Scientific, Waltham, MA, USA). Cells were treated with PEA-Hyb-np_Cum_ at a final PEA concentration of 100 μM for 14 h.

Following incubation, cells were fixed with 3% (*w*/*v*) paraformaldehyde for 15 min at room temperature. After fixation, cells were stained with Hoechst 33342 to visualize nuclei, washed with PBS, and imaged using a Leica SP8 confocal microscope (DMIRE2; Leica Microsystems GmbH, Wetzlar, Germany). Imaging was performed with a 63× Plan-Apochromat oil immersion objective (Leica Microsystems GmbH, Wetzlar, Germany). Excitation was achieved using a 405 nm laser for Hoechst and a 470 nm laser for Coumarin-6. Fluorescence images were acquired and processed using the instrument’s proprietary software (.LasX, version 3.3.0).

### 2.14. Statistical Analysis

Data were analyzed using one-way analysis of variance (ANOVA). Statistical significance was set at *p* < 0.05. Results are reported as follows: *p* < 0.05 (*), *p* < 0.01 (**), *p* < 0.0002 (***), and *p* < 0.0001 (****).

## 3. Results and Discussion

### 3.1. Nanoparticle Formulation and Characterization

Encapsulating lipophilic molecules such as PEA within PLGA nanoparticles is inherently challenging due to the limited affinity between highly hydrophobic compounds and the PLGA polymer matrix. Initial attempts to formulate PEA-loaded nanoparticles using a conventional single-emulsion solvent evaporation method, where PEA and PLGA were co-dissolved in dichloromethane and emulsified in an aqueous phase, resulted in suboptimal particles with a mean diameter of about 386 ± 15 nm, a PDI of 0.384 ± 0.043 and a negative surface charge of −20.6 ± 1.7 mV (Table 2). Encapsulation efficiency (EE) was just 3.9 ± 0.2%, likely due to the low affinity of PEA with the PLGA matrix, resulting in its partial precipitation and aggregation outside the particle core. This is consistent with the literature indicating that PLGA nanoparticles are poorly suited to encapsulate small lipophilic drugs without appropriate formulation strategies [20].

To improve encapsulation, stearic acid was introduced into the organic phase. Stearic acid is a biocompatible saturated fatty acid capable of forming non-covalent hydrophobic interactions with lipid molecules such as PEA, enhancing their retention within the nanoparticle core [7,18]. This modified formulation, PEA/Ste-PLGA-np, showed a significant increase in EE to 31.3 ± 1.6% and a pronounced size reduction to 128 ± 8 nm, along with a more negative zeta potential (−33.9 ± 0.4 mV), suggesting increased surface charge density due to lipid content. However, physical instability was noted, with the rapid appearance (less than 30 min) of floating aggregates, likely reflecting partial lipid crystallization or incompatibility within the polymer matrix.

To address this inconvenience, Gelucire^®^ 50/13 was incorporated as a lipid-based surfactant and stabilizing agent. Gelucire^®^ is an amphiphilic excipient composed of mono-, di-, and triglycerides and polyethylene glycol esters of fatty acids. It is widely recognized for its biocompatibility and surfactant properties and is generally regarded as safe (GRAS) by regulatory authorities [21]. Moreover, Gelucire^®^ has been shown to inhibit P-glycoprotein-mediated drug efflux, potentially enhancing intracellular uptake of encapsulated compounds [22]. Initially, Gelucire^®^ was individually added to the PEA-PLGA nanoparticles at a 1:1 ratio relative to PLGA, based on preliminary formulation tests. The resulting PEA/Gel-PLGA-np formulation exhibited an average particle size of 183 ± 30 nm and a PDI of 0.280 ± 0.019, indicating moderate uniformity, comparable to the PEA/Ste-PLGA-np formulation. The surface charge measured −25.3 ± 1.3 mV, slightly less negative than that of the stearic acid-based np, possibly due to partial surface coverage by Gelucire^®^ molecules. Although encapsulation efficiency improved significantly to approximately 56%, the physical stability profile remained similar to that of PEA/Ste-PLGA-np, with observable aggregation over time. A combined strategy was then explored to harness the stabilizing and solubilizing benefits of both stearic acid and Gelucire^®^ in a single hybrid formulation. These optimized additions yielded a stable hybrid nanoparticle system (PEA-Hyb-np) with a size of 146 ± 7 nm, PDI of 0.268 ± 0.051, and EE of 79.1 ± 0.1%. In this optimized hybrid preparation, the Drug Loading was 790 μg/mL corresponding to 44.2 mg/g of particles. This latter value was determined based on the recovery of approximately 18 mg of particles after vacuum centrifuge drying of 1 mL of the particle suspension.

To gain further insight into the variations in the above-described characteristics among the different samples, several possible phenomena were considered. Among these, it is well established that fatty acids can participate in the hydrophobic ion pairing (HIP) phenomenon. This process has attracted significant research interest, as evidenced by several comprehensive reviews on the topic [23,24,25]. However, for such interactions to occur, the presence of a basic molecule bearing at least one protonable amine group is generally required. PEA (Figure S2) does not contain any protonable amine groups; instead, it features an amide group, which does not undergo protonation. Therefore, it is reasonable to assume that the increased encapsulation efficiency (EE) observed in PEA-Hyb-np, compared to both PEA-PLGA-np and PLGA np containing a single lipid component, can be attributed to hydrophobic interactions among the three lipid components (stearic acid, Gelucire, and PEA). Nevertheless, the involvement of HIP interactions cannot be entirely ruled out and will be further investigated in future studies.

As regards suspension stability, no macroscopic aggregation was observed even after 24 h at room temperature, indicating excellent colloidal stability. These improvements point to the successful formation of a polymer-lipid hybrid structure, where the drug and excipients interact synergistically to promote efficient encapsulation and structural homogeneity.

Interestingly, unloaded hybrid nanoparticles (U-Hyb-np) exhibited no significant variation in surface charge (−30.7 ± 0.8 mV), but a slightly larger average diameter (167 ± 6 nm) and higher PDI (0.324 ± 0.113) compared to the PEA-loaded counterpart. This difference may be explained by the role of PEA in facilitating tighter molecular packing through hydrophobic interactions with stearic acid and Gelucire^®^, thereby contributing to the formation of more compact and stable nanoparticles, as discussed above. In the absence of PEA, the internal structure may be more disordered, with less cohesive forces acting during nanoparticle assembly, resulting in increased particle size and heterogeneity.

### 3.2. Characterization of the Optimized PEA-Hyb-np

#### 3.2.1. Morphology Study

**Figure 1:**
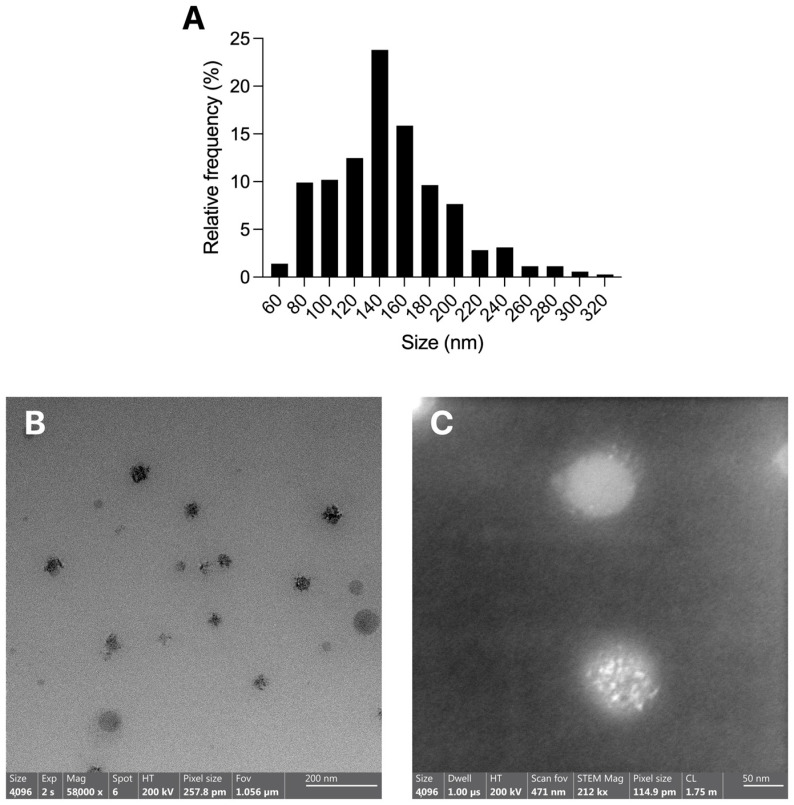
(**A**) Histogram of the particle size distribution of PEA-Hyb-np from Transmission electron microscopy (TEM) images; (**B**) TEM images of PEA-Hyb-np at low magnification; (**C**) High magnification image in STEM mode showing internal heterogeneous structure.

Figure 1A reported the histogram of the particle size distribution of PEA-Hyb-np obtained from TEM images (Figure 1B,C and Figure S3). Data show that the most frequent size (approximately 140 nm) matches the values measured with the Nanosizer and reported in Table 2. Figure 1B, illustrates PEA-Hyb-np with a predominantly spherical shape with well-defined contours with a diameter of about 100 nm or smaller.

A more detailed morphological inspection was carried out using scanning TEM (STEM) mode, which enables enhanced structural analysis of the nanoparticles through the use of a focused electron beam that scan across the sample, providing high-resolution imaging and compositional contrast. Indeed, Figure 1C, in addition to the higher magnification, some particles revealed internal features suggestive of a “polynucleated” or compartmentalized inner structure. Such morphology is likely due to the hybrid composition of the nanoparticles, where lipid components, namely stearic acid and Gelucire^®^ 50/13, are incorporated within the polymeric matrix. This heterogeneous internal architecture is a hallmark of lipid-polymer hybrid systems, in which phase separation between the lipid and polymer domains can occur during nanoparticle formation [26]. The darker electron-dense regions observed may correspond to lipid-enriched microdomains, whereas the surrounding lighter areas are attributable to the PLGA-rich matrix.

The presence of this polynucleated structure suggests a successful integration of lipidic materials into the polymeric scaffold, likely contributing to the high encapsulation efficiency and colloidal stability described previously. Furthermore, such nanostructured internal organization can be advantageous for providing multiple microenvironments within the nanoparticle that may influence diffusion rates and release kinetics [14].

#### 3.2.2. Stability Assessment

**Figure 2:**
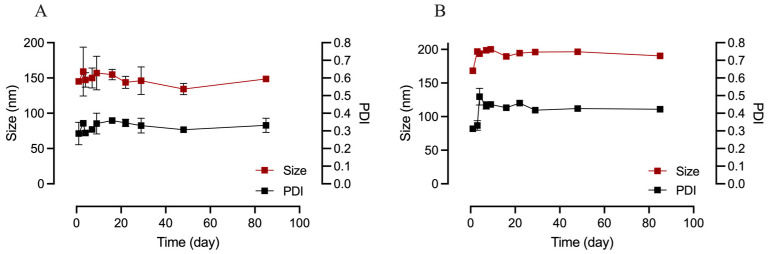
Stability of PEA-Hyb-np (**A**) and U-Hyb-np (**B**) Size and polydispersity index (PDI) measured over 80 days of storage at 4 °C.

Given the lipidic component in the hybrid formulation, a stability assay was conducted to evaluate whether lipid expulsion from the polymeric matrix could occur over time during storage in suspension. This analysis is critical to ensure consistent performance and reproducibility in drug delivery applications. To this end, both PEA-Hyb-np and U-Hyb-np were monitored over an 80-day period for variations in particle size and PDI (Figure 2).

Throughout the observation period, the size of PEA-Hyb-np fluctuate around a value of 140 nm, with a PDI around 0.30, indicating good colloidal stability and uniform size distribution. In contrast, U-Hyb-np showed slightly larger particle sizes (approximately 170 nm), along with a gradual increase in PDI over time. This suggests a tendency toward colloidal destabilization in the absence of PEA.

The greater stability of the drug-loaded nanoparticles may be attributed to the interaction between PEA and the lipid-polymer matrix, which likely contributes to structural reinforcement and prevents phase separation. In lipid-polymer hybrid systems, the inclusion of hydrophobic drug molecules can enhance matrix cohesion and reduce lipid leakage or aggregation phenomena [14,27].

#### 3.2.3. In Vitro PEA Release from Hybrid Nanoparticles

**Figure 3:**
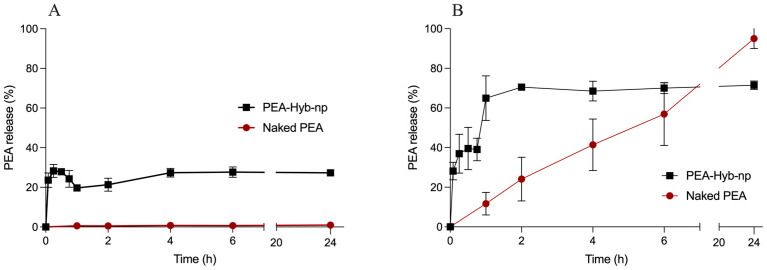
(**A**) In vitro release profile of Naked PEA and PEA-Hyb-np in phosphate-buffered saline (PBS, pH 7.4) at 37 °C; (**B**) Comparative release profile of Naked PEA and PEA-Hyb-np in 2% (*w*/*v*) sodium taurocholate solution at 37 °C. Data represent mean ± SD (*n* = 6).

The release profile of PEA from PEA-Hyb-np was evaluated using a dialysis method and compared to the dissolution behavior of Naked PEA. In a physiological medium consisting of phosphate-buffered saline (PBS, pH 7.4), Naked PEA exhibited negligible dissolution, with concentrations remaining below detectable limits throughout the analysis period (Figure 3A). In contrast, PEA released from the hybrid nanoparticles displayed a detectable profile, characterized by an initial burst release of approximately 20% within the first few minutes, followed by a plateau phase that extended up to 24 h.

These findings clearly indicate that Naked PEA is poorly soluble in aqueous physiological conditions, in agreement with its high lipophilicity, whereas its encapsulated form demonstrates improved apparent solubility. This evidence is likely facilitated by the presence of stearic acid and Gelucire within the formulation, which may enhance drug dispersion, as previously observed.

To better elucidate the solubility and release dynamics, a non-physiological medium, i.e., 2% (*w*/*v*) sodium taurocholate solution, was employed. This surfactant-rich medium provides enhanced solubilization capacity for lipophilic drugs such as PEA, reaching an apparent solubility of approximately 140 μg/mL. Under these conditions, both Naked PEA and PEA-Hyb-np were tested using an equivalent drug concentration of 90 μg/mL, deliberately kept below the solubility limit to avoid supersaturation effects.

In sodium taurocholate solution (Figure 3B) even if sink conditions are still not reached, Naked PEA exhibited a slow and gradual dissolution, dissolving completely after 24 h. In contrast, PEA-Hyb-np showed a significantly faster dissolution rate, characterized by an initial burst effect of 70% within the first 2 h, followed by a sustained release phase over the remaining 22 h.

The results obtained using this non physiological medium suggest that while a fraction of PEA is loosely associated with the nanoparticle surface and rapidly diffuses upon contact with the medium, the remaining drug is more deeply embedded within the polymeric matrix. This entrapped fraction is likely to be released gradually in vivo through the hydrolytic degradation of PLGA, providing a prolonged therapeutic effect.

The biphasic drug release profile may represent a clinical advantage. On the one hand, the initial phase can increase the solubility and consequently the bioavailability of the molecule, potentially triggering the therapeutic effect. On the other hand, the gradual release observed in the second phase may help sustain the therapeutic action over a prolonged period. In this context, the release profile of PEA observed in this study may align well with the desired pharmacological behavior for chronic inflammatory conditions

Different strategies have been developed to enhance the bioavailability of PEA. These include micronized and ultramicronized forms, which improve the dissolution rate and gastrointestinal absorption compared with Naked PEA [28], as well as water-dispersible formulations, designed to promote aqueous dispersion and increase systemic exposure after oral administration. Recent pharmacokinetic studies have confirmed that both micronized and water-dispersible PEA achieve higher plasma concentrations than Naked PEA [29]. Some of these formulations provide further evidence that improving PEA dispersion in aqueous environments can significantly enhance its bioavailability [5,30]. These considerations further support the rationale underlying the design of nanoparticles, which were formulated to achieve comparable dispersion and release properties, albeit intended for a different route of administration than the oral formulations commonly described in the literature and available on the market.

#### 3.2.4. Thermal Analysis

**Figure 4:**
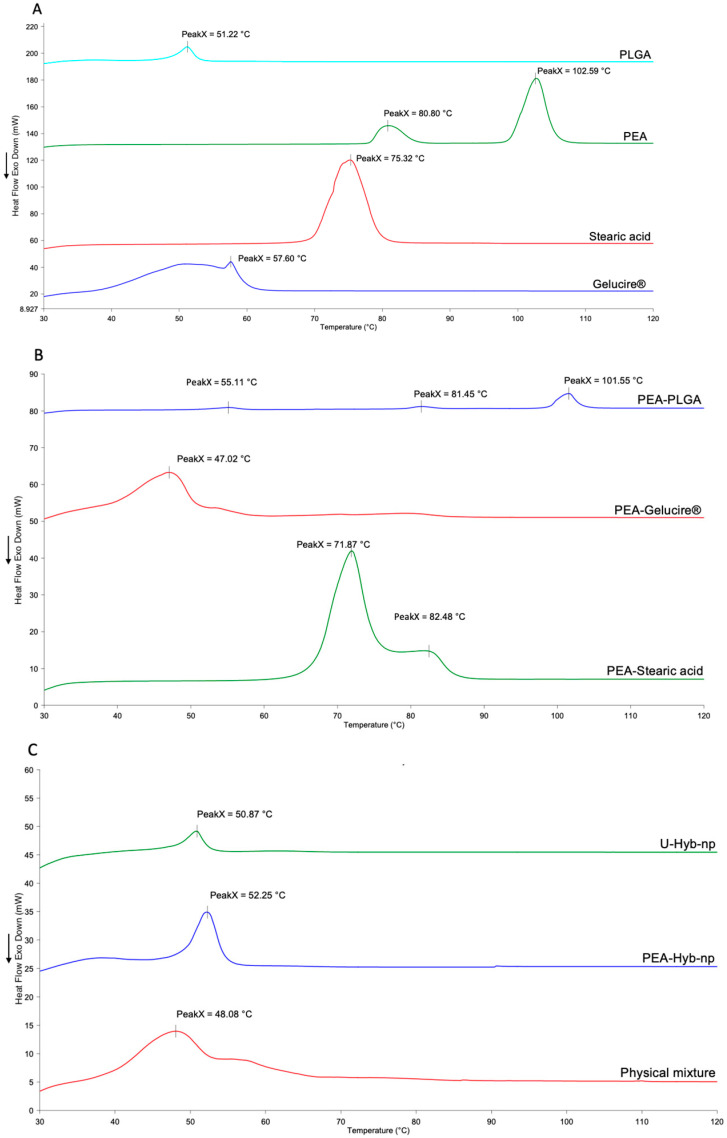
DSC thermogram of (**A**) raw materials; (**B**) binary physical mixtures of PEA-PLGA, PEA-Gelucire, and PEA-Stearic Acid; (**C**) U-Hyb-np, PEA-Hyb-np, and physical mixture of PEA-PLGA-Stearic acid-Gelucire.

The thermal behavior of Naked PEA, PEA-Hyb-np, U-Hyb-np, and physical mixtures of PEA with individual nanoparticle components is presented in Figure 4. The physical mixtures were prepared using the same weight ratios employed in the nanoparticle formulations.

As shown in Figure 4A, PLGA exhibited a single endothermic event corresponding to its glass transition temperature (Tg) at approximately 51.2 °C, consistent with its amorphous, random copolymer nature and the absence of crystalline melting peaks. Naked PEA displayed two endothermic peaks at 80.8 °C and 102.6 °C, indicative of its polymorphic crystalline structure. Stearic acid showed a single, well-defined melting peak at 75.3 °C. The DSC thermogram of Gelucire^®^ 50/13 exhibited a broad, poorly defined endothermic peak at 57.8 °C, reflecting its composition as a mixture of mono-, di-, and triglycerides as well as polyethylene glycol esters of fatty acids.

The DSC thermograms of binary physical mixture of PEA with the individual components are reported in Figure 4B. The mixture between PEA and PLGA exhibited endothermic events characteristic of both components, confirming the persistence of PEA in its crystalline form when associated solely with the polymer even if the intensity of PEA’s peaks was reduced as a consequence of the mixing process. Interestingly, the physical mixture of PEA with Gelucire (PEA:Gelucire weight ratio 1:8) did not display the characteristic endothermic peaks of PEA: only the Gelucire melting peak was present, suggesting that PEA was fully solubilized and amorphized within the lipid matrix upon melting. This observation supports the hypothesis that similar molecular interactions occur during nanoparticle formulation, when both components are solubilized in a shared solvent phase. A comparable trend was observed for the physical mixture of PEA with stearic acid at a 1:1 weight ratio: although one of the PEA polymorphic peaks remained visible, its intensity was significantly reduced. This partial amorphization is in agreement with previous findings reported for solid lipid nanoparticles (SLNs) prepared using a PEA:stearic acid ratio of 1:2.5 where partial disappearance of the drug’s melting peaks was observed [7].

In Figure 4C, DSC thermograms of U-Hyb-np, PEA-Hyb-np, and a physical mixture of all components are shown. Notably, the thermogram of PEA-Hyb-np did not exhibit any PEA-associated endothermic peaks, apart from the PLGA glass transition. This absence of PEA melting signals confirms the transition of the drug from a crystalline to an amorphous state within the nanoparticle matrix. The absence of PEA in crystalline form in the PEA-Hyb-np is further confirmed by the lack of an exothermic transition in the cooling curves of the loaded nanoparticles (Figure S4).

These thermal analysis results indicate that PEA exists in an amorphous form both within the hybrid nanoparticles and in physical mixtures with lipid excipients under formulation-relevant conditions.

The amorphization of PEA within the hybrid nanoparticles offers key biopharmaceutical advantages. Amorphous forms lack the crystalline lattice, resulting in higher apparent solubility and faster dissolution rates, which are crucial for improving the bioavailability of poorly water-soluble drugs like PEA [31]. The lipidic excipients (Gelucire^®^ and stearic acid) likely contribute to both drug amorphization and physical stabilization within the nanoparticle matrix, preventing recrystallization over time [32]. This enhanced solubilization is consistent with the improved in vitro release observed in aqueous media, where free PEA was poorly soluble. Overall, converting PEA to its amorphous form within a hybrid nanocarrier system represents a strategic approach to overcome its pharmacokinetic limitations and improve therapeutic performance.

#### 3.2.5. FT-IR

**Figure 5:**
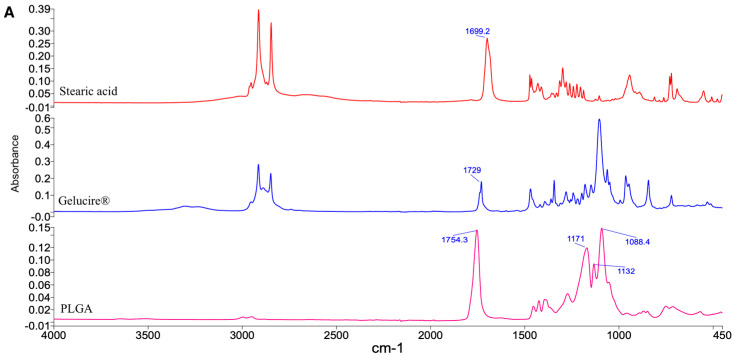

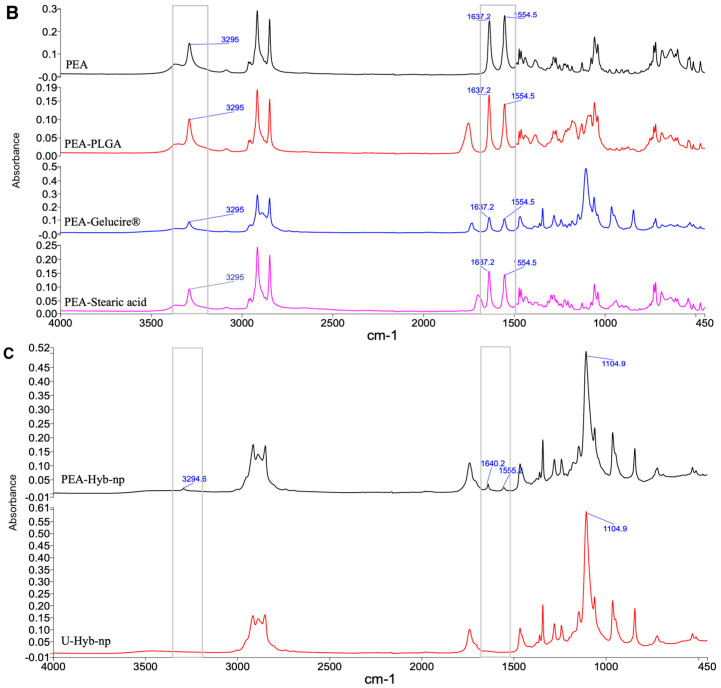
IR spectra of (**A**) raw materials, (**B**) Naked PEA and binary physical mixtures of PEA-PLGA, PEA-Gelucire, and PEA-Stearic Acid; (**C**) PEA-Hyb-np and U-Hyb-np.

Fourier-transform infrared (FT-IR) spectra were acquired for raw excipients (Figure 5A), Naked PEA, binary physical mixtures of PEA with PLGA, Gelucire^®^, and stearic acid (Figure 5B), as well as for the PEA-Hyb-np and their unloaded counterparts, U-Hyb-np (Figure 5C).

The FT-IR spectrum of Naked PEA displayed sharp absorption bands at 1637 cm^−1^ and 1555 cm^−1^, corresponding to the stretching vibrations of the carbonyl (C=O) group and N–H binding of the amide, respectively. Moreover, in the range between 3000 and 3500 cm^−1^ typical peaks indicating vibration of -OH groups are visible. All these characteristic bands are consistent with the molecular structure of PEA and have been previously reported in the literature [33].

Importantly, no new peaks were detected in the spectra of the physical mixtures between PEA and the individual excipients, compared to the single spectra of raw excipients, suggesting the absence of chemical interactions or covalent bond formation. The preservation of the characteristic peaks of both PEA and PLGA implies a physical rather than chemical interaction between the components.

However, in the PEA-Hyb-np formulation, the intensity of the PEA-specific bands was significantly reduced compared to the physical mixtures. This attenuation of the characteristic peaks suggests a loss of long-range molecular order, indicating a reduction in crystallinity. When considered alongside the DSC data, which also demonstrated the absence of PEA’s melting endotherms, these findings confirm that PEA is present in an amorphous state within the nanoparticle matrix.

The preservation of PEA’s key functional group peaks in the FT-IR spectra further supports the conclusion that the drug maintains its chemical integrity throughout the encapsulation process. Together, these results indicate successful incorporation of PEA into the hybrid nanoparticles, with no evidence of degradation or chemical modification during formulation.

This molecular dispersion of amorphous PEA within the polymer-lipid matrix is advantageous, as it facilitates improved solubilization and dissolution performance, consistent with findings reported for other hydrophobic compounds encapsulated in hybrid nanosystems [31,32].

#### 3.2.6. XRD Scanning Analysis

**Figure 6:**
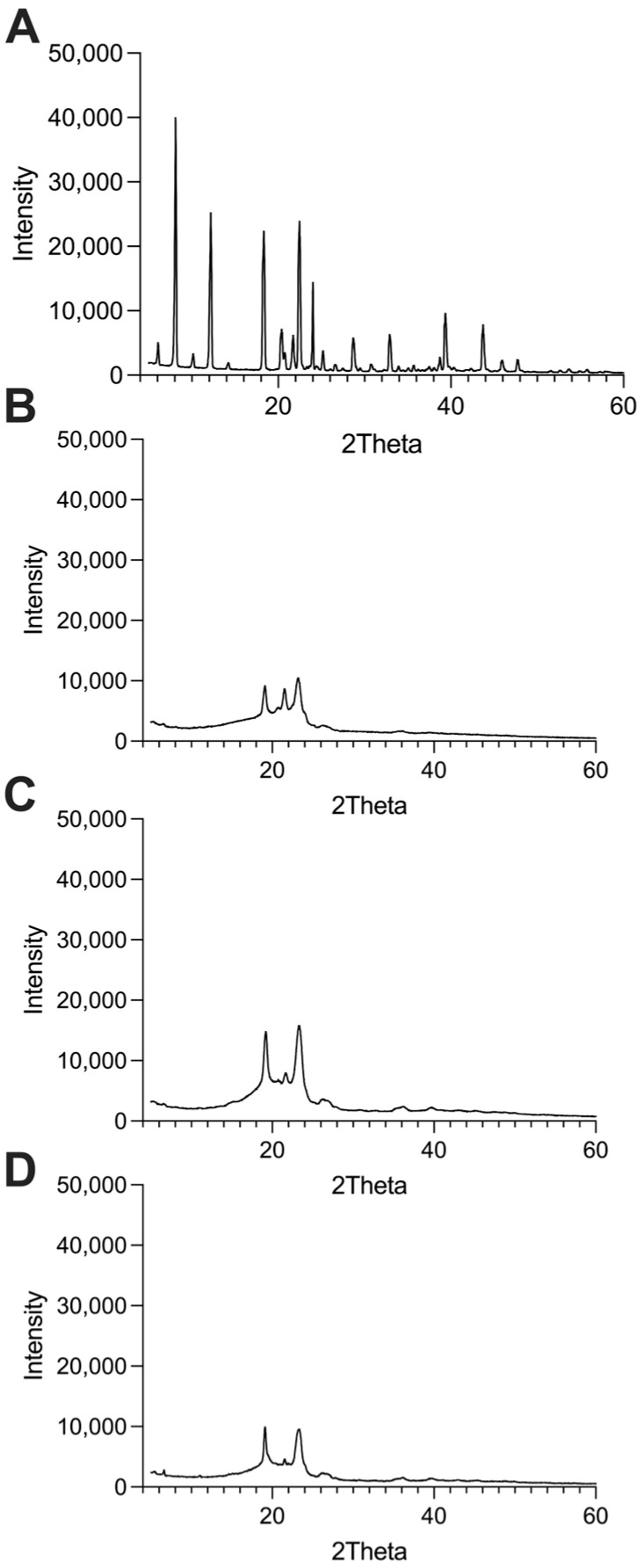
XDR spectra of (**A**) Naked PEA, (**B**) physical mixtures of components; (**C**) PEA-Hyb-np, and (**D**) U-Hyb-np.

The XRD patterns are shown in Figure 6. Multiple characteristic crystalline diffraction peaks were observed for PEA, with representative peaks at 8.1°, 12.2°, 18.3–18.4°, and 22.5°, all exhibiting intensities above 20 k counts. In contrast, these characteristic peaks were absent in the PEA-Hyb-np samples, indicating the lack of crystalline PEA. The diffraction pattern of the loaded nanoparticles was, in fact, practically superimposable on that of the unloaded ones. Notably, even in the case of the physical mixture, no peaks attributable to crystalline PEA were detected, suggesting that PEA became amorphous due to the formation of drug–lipid interactions with stearic acid and/or Gelucire through non-covalent bonds.

### 3.3. In Vitro Cell Analyses

#### 3.3.1. Cytotoxicity Assessment

**Figure 7:**
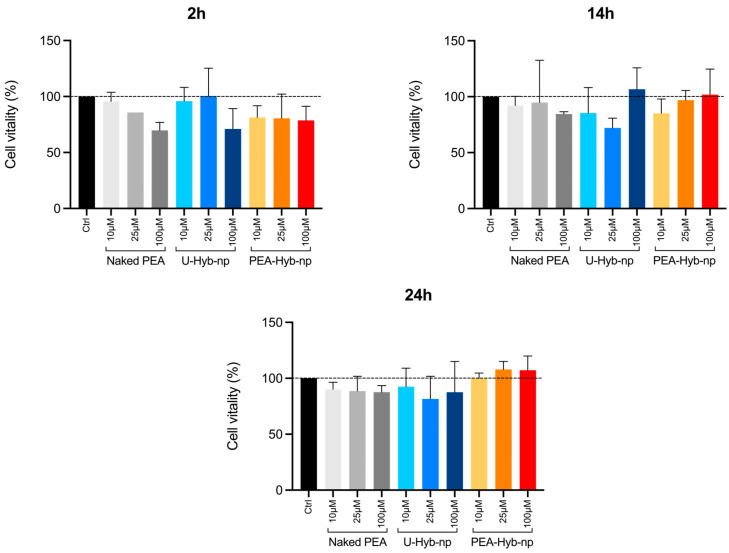
Cytotoxicity of Naked PEA, U-Hyb-np, and PEA-Hyb-np on C2C12 cells at PEA concentrations of 10, 25, and 100 µM after 2, 14, and 24 h of incubation, expressed as percentage cell viability.

MTT assays were conducted to evaluate the cytotoxic potential of Naked PEA, PEA-Hyb-np and U-Hyb-np on C2C12 cells. Results indicated no significant cytotoxicity for any of the tested samples across all concentrations and incubation times, with cell viability consistently remaining high (Figure 7). Overall, all samples exhibited good biocompatibility and minimal cytotoxic effects. The cytotoxicity results demonstrated that all tested samples (PEA-Hyb-np, U-Hyb-np, and Naked PEA) were biocompatible and non-cytotoxic under the conditions tested. Although a trend of higher viability was observed in cells treated with PEA-Hyb-np compared to Naked PEA and U-Hyb-np, these differences were not statistically significant. However, the trend observed may be attributable to the prolonged release profile of PEA provided by the nanoparticle formulation, potentially reducing cellular stress and improving cell tolerance. These findings underscore the suitability of PEA-Hyb-np for potential therapeutic applications, highlighting the possible advantages of nanoparticle encapsulation strategies for enhancing both bioactivity and compatibility of active compounds compared to their Naked counterparts [20].

#### 3.3.2. Cellular Uptake

**Figure 8:**
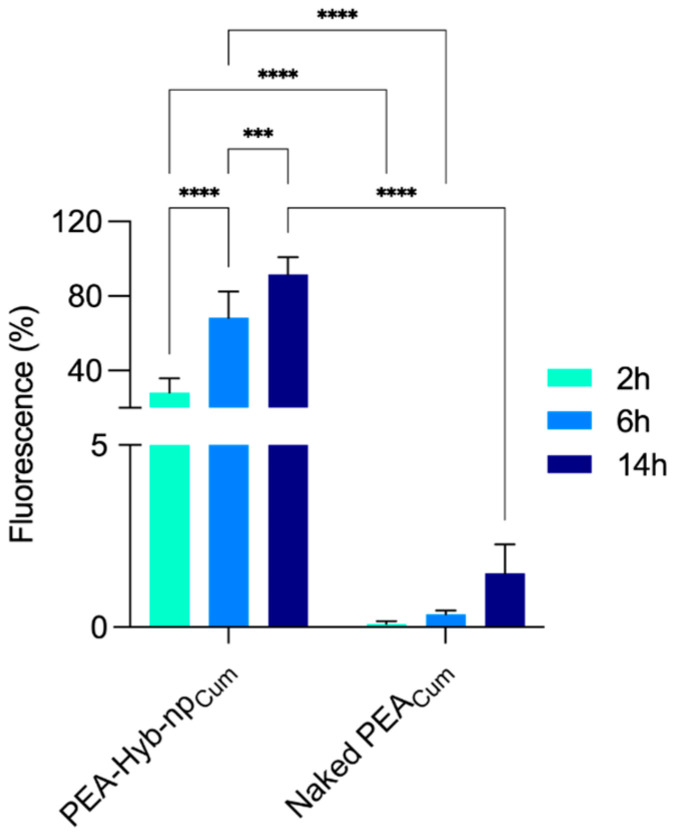
Flow cytometric analysis of C2C12 cells following 2, 6, and 14 h of incubation with PEA-Hyb-np_Cum_ and Naked PEA_Cum_ at a concentration of 100 µM. Statistical significance is indicated as *** (*p* = 0.0004); **** (*p* < 0.0001).

**Figure 9:**
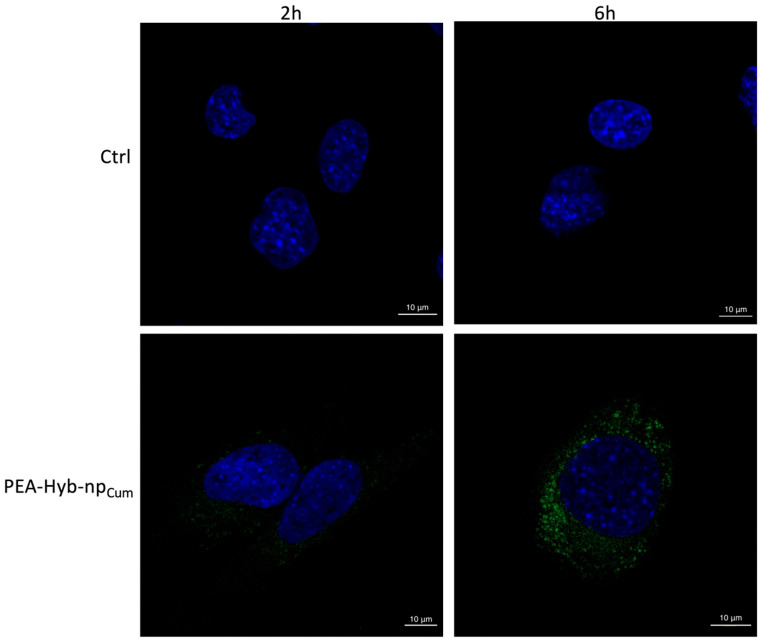
Representative confocal fluorescence images of C2C12 cells after 2 and 6 h of exposure to PEA-Hyb-np_Cum_. Nanoparticles were labeled with coumarin-6 (green channel), and cell nuclei were stained with DAPI (blue channel). Untreated cells were used as control.

Flow cytometric analyses indicated that nanoparticles were efficiently internalized by C2C12 cells, with significantly higher internalization rates when compared to Naked PEA_Cum_, at all analyzed incubation times (2, 6, and 14 h) (*p* < 0.0001) (Figure 8). In particular, after 14 h of incubation, the fluorescence signal associated with Naked PEA remained around 2%, whereas PEA-Hyb-np exhibited a markedly higher uptake, exceeding 80%. Confocal microscopy further confirmed these results, revealing intense intracellular green fluorescence associated with PEA-Hyb-np_Cum_. Observations at 2 and 6 h post-incubation demonstrated clear nanoparticle uptake within cytoplasmic compartments, while untreated control cells showed no fluorescence (Figure 9).

Flow cytometry and confocal microscopy data collectively indicate efficient uptake of PEA-Hyb-np by C2C12 myoblasts. Confocal microscopy reveals a predominantly cytoplasmic distribution of the nanoparticles, consistent with internalization via the endocytic pathway. Similarly to other polymeric nanoparticles, and considering that PLGA serves as the outer matrix embedding the lipid components, the main mechanism involved is clathrin-mediated endocytosis. Following internalization, the nanoparticles are released into the cytosol through endosomal escape, which occurs via temporary destabilization of the endosomal membrane, in agreement with previous reports on PLGA-based systems [34,35]. These findings support the potential of the hybrid nanoparticle platform to enhance targeted cellular delivery and cytoplasmic release of encapsulated therapeutic agents such as PEA [36].

## 4. Conclusions

This study demonstrated the successful development of hybrid PLGA-lipid nanoparticles for the encapsulation of PEA, overcoming key biopharmaceutical limitations such as poor solubility and low bioavailability through the rational inclusion of lipid excipients like stearic acid and Gelucire^®^ 50/13, which enabled the formation of stable, small, and polynucleated particles with high encapsulation efficiency. The formulation strategy enabled the transition of PEA into an amorphous state, confirmed by DSC and FT-IR analyses, which contributed to enhanced dissolution and potential bioactivity. The hybrid system exhibited good colloidal stability over time, with no evidence of lipid expulsion from the polymeric matrix. In vitro assays confirmed the safety and biocompatibility of the nanoparticles, with no significant cytotoxic effects on C2C12 cells. Moreover, flow cytometry and confocal microscopy showed efficient and time-dependent cellular uptake of the nanoparticles, confirming their potential for targeted intracellular delivery in skeletal muscle cells. Collectively, these findings demonstrate the efficacy and biocompatibility of the PEA-loaded hybrid PLGA-lipid nanoparticle platform, providing a solid basis for future in vivo studies and potential clinical translation. Although this work was conducted exclusively on C2C12 myoblasts as a proof-of-concept, several challenges must be addressed to translate these results to in vivo systems. Biodistribution, pharmacokinetics, and potential immunological responses will be critical factors influencing therapeutic performance. Parameters such as nanoparticle size, surface charge, and composition are expected to affect circulation, tissue accumulation, and clearance. Thorough in vivo investigations will therefore be essential to fully assess the translational potential of this delivery system for muscle-targeted therapies.

## Figures and Tables

**Table 1:** Summary of all prepared formulations with their components.

Sample	Organic Phase (in 3 mL of DCM)					Aqueous Phase (10 mL)
	Stearic Acid (mg)	Gelucire (mg)	PLGA (mg)	PEA (mg)	Span85 (mg)	Pluronic F68 (%, *w*/*v*)
PEA-PLGA-np	-	-	80	10	120	0.8
PEA/Ste-PLGA-np	10	-	80	10	120	0.8
PEA/Gel-PLGA-np	-	80	80	10	120	0.8
PEA-Hyb-np	10	80	80	10	120	0.8
U-Hyb-np	10	80	80	-	120	0.8

**Table 2:** Characterization of nanoparticles during optimization process, including measurements of size, polydispersity index (PDI), surface charge (Z-potential), PEA encapsulation efficiency (EE).

Sample	Size (nm ± SD)	PDI (± SD)	Z-Potential (mV ± SD)	EE (% ± SD)
PEA-PLGA-np	386 ± 15	0.384 ± 0.043	−20.6 ± 1.7	3.9 ± 0.2
PEA/Ste-PLGA-np	128 ± 8	0.238 ± 0.010	−33.9 ± 0.4	31.3 ± 1.6
PEA/Gel-PLGA-np	183 ± 30	0.280 ± 0.019	−25.3 ± 1.3	56.8 ± 0.7
PEA-Hyb-np	146 ± 7	0.268 ± 0.051	−29.6 ± 0.8	79.1 ± 0.1
U-Hyb-np	167 ± 6	0.324 ± 0.113	−30.7 ± 0.8	-

## Data Availability

The data supporting the conclusions of this article will be made available by the authors on request.

## Supplementary Materials

The following supporting information can be downloaded at: https://www.mdpi.com/article/10.3390/pharmaceutics17111412/s1, Figure S1. Draw sketch for preparation method of PEA-Hyb-np; Figure S2. Palmitoylethanolamide (PEA) structure; Figure S3. Representative electron microscopy images of PEA-Hyb-np. (A) TEM image showing the nanoparticle morphology; (B) STEM image acquired using the STEM detector, highlighting the internal structure; Figure S4. DSC heating and cooling curves of loaded and unloaded nanoparticles, Physical mixture and Naked PEA

## Abbreviations

The following abbreviations are used in this manuscript:

DCM	Dichloromethane
DL	Drug Loading
DLS	Dynamic Light Scattering
DMEM	Dulbecco’s Modified Eagle Medium
DMSO	Dimethyl sulfoxide
DSC	Differential scanning calorimetry
EE	Encapsulation Efficiency
EMA	European Medicines Agency
FBS	Fetal bovine serum
FDA	Food and Drug Administration
FT-IR	Fourier-transform infrared
GRAS	Generally regarded as safe
HAADF	High-angle annular dark-field
HPLC	High-performance liquid chromatography
MTT	Thiazolyl Blue Tetrazolium Bromide
MWCO	Molecular weight cut-off
P/S	Penicillin–streptomycin
PBS	Phosphate-buffered saline
PDI	Polydispersity index
PEA	Palmitoylethanolamide
PEA-Hyb-np	PEA-loaded hybrid nanoparticles
PEA-PLGA-np	PEA-loaded PLGA nanoparticle
PEA/Gel-PLGA-np	PEA/Gelucire-loaded PLGA nanoparticle
PEA/Ste-PLGA-np	PEA/Stearic acid-loaded PLGA nanoparticle
PLGA	Poly(lactic-co-glycolic acid)
PPAR-α	Peroxisome proliferator-activated receptor alpha
TEM	Transmission Electron Microscopy
U-Hyb-np	Unloaded hybrid nanoparticles
