# Supplementary material for: Investigating Hybrid PLGA-Lipid Nanoparticles as an Innovative Delivery Tool for Palmitoylethanolamide to Muscle Cells
Source: Pharmaceutics. 2025 Oct 30;17(11):1412. doi: 10.3390/pharmaceutics17111412 (PMC12655583; doi:10.3390/pharmaceutics17111412)
Supplement: Supplementary file 1 [file pharmaceutics-17-01412-s001.zip › pharmaceutics-3875446-supplementary.pdf]

Electronic Supplementary information

Article

Investigating Hybrid PLGA-Lipid Nanoparticles as an Innovative Delivery Tool for Palmitoylethanolamide to muscle cells

Eleonora Maretti[1], Susanna Molinari[2], Sonia Partel[1], Beatrice Recchia[2], Cecilia Rustichelli[1], and Eliana Leo[1*]

[1] Department of Life Sciences, University of Modena and Reggio Emilia, Via Campi 103, 41125 Modena, Italy; eleonora.maretti@unimore.it (E.M.); partelsonia@gmail.com (S.P.); cecilia.rustichelli@unimore.it (C.R.).
[2] Department of Life Sciences, University of Modena and Reggio Emilia, Via Campi 287, 41125 Modena, Italy; susanna.molinari@unimore.it (S.M.); beatricerecchia9@gmail.com (B.R.).
* Correspondence: eliana.leo@unimore.it (E.L.); Tel.:+39 059 2058558

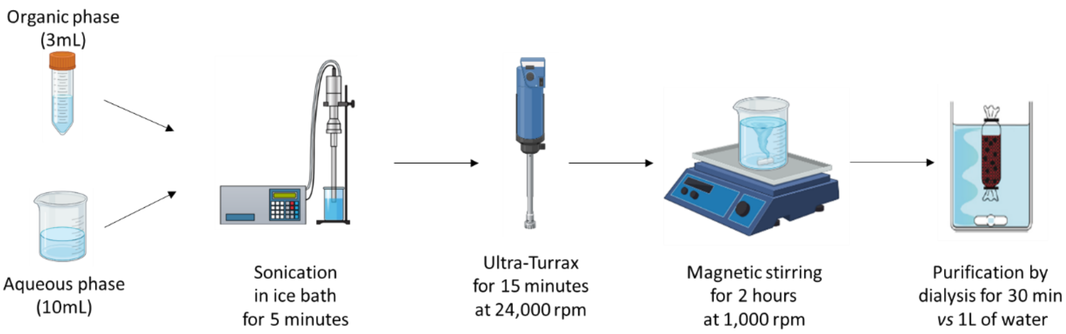

Figure S1. Draw sketch for preparation method of PEA-Hyb-np

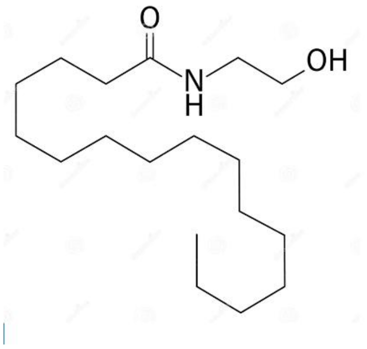

Figure S2. Palmitoylethanolamide (PEA) structure

A

B

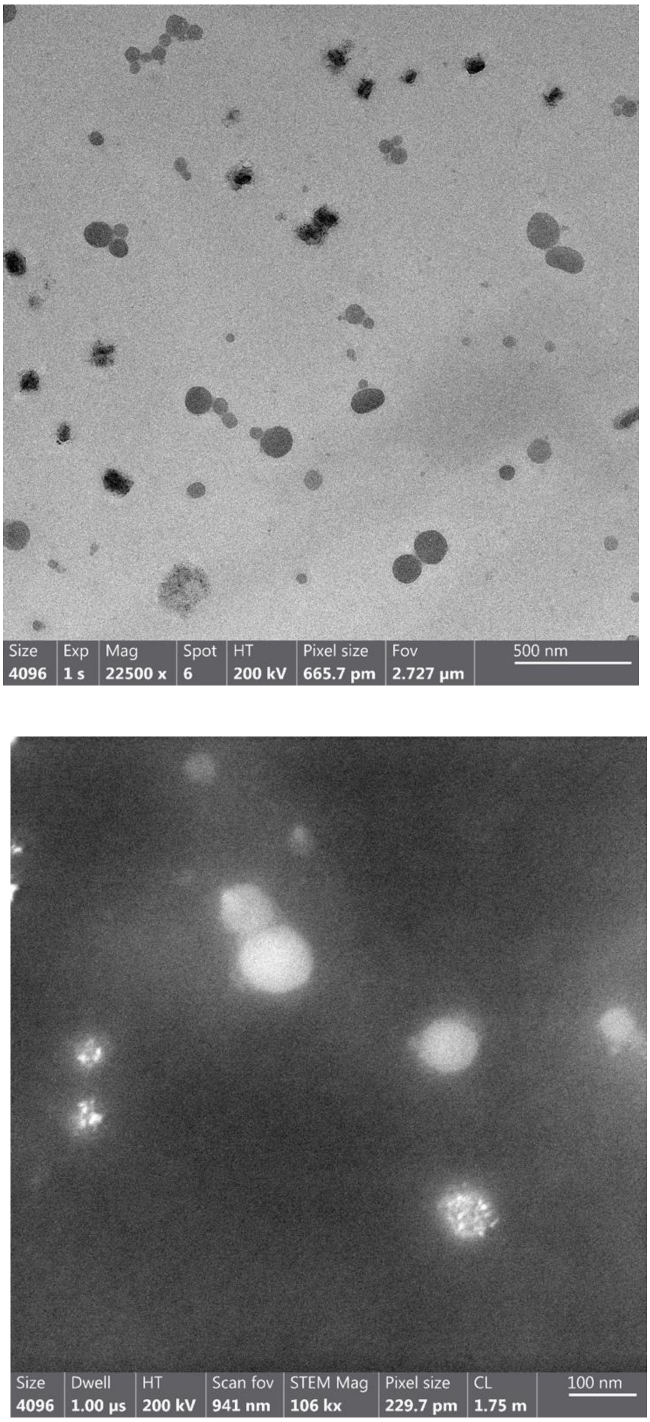

Figure S3. Representative electron microscopy images of PEA-Hyb-np. (A) TEM image showing the nanoparticle morphology; (B) STEM image acquired using the STEM detector, highlighting the internal structure

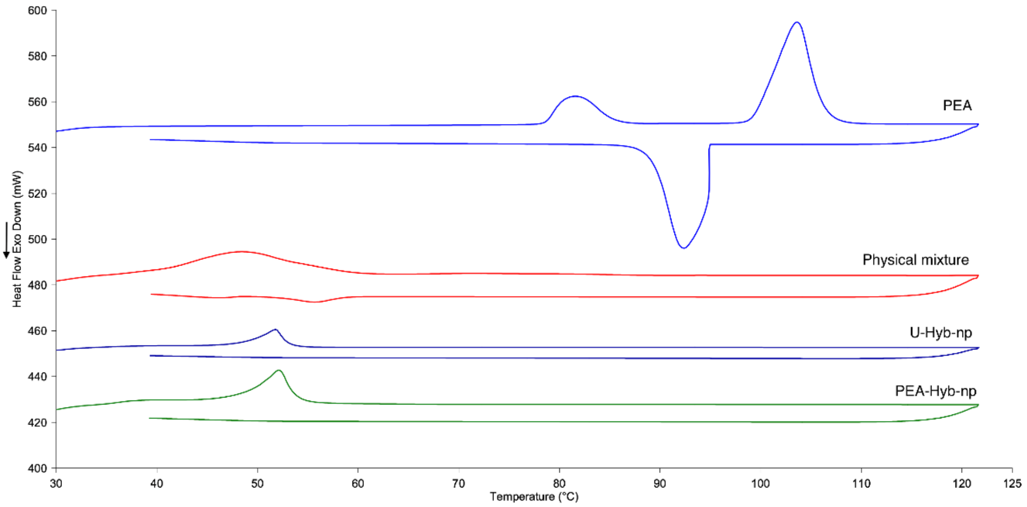

Figure S4. DSC heating and cooling curves of loaded and unloaded nanoparticles, Physical mixture and Naked PEA
